# Enhancement of Doppler spectroscopy to transverse direction by using optical vortex

**DOI:** 10.1038/s41598-023-42517-z

**Published:** 2023-09-16

**Authors:** Hiroki Minagawa, Shinji Yoshimura, Kenichiro Terasaka, Mitsutoshi Aramaki

**Affiliations:** 1 College of Industrial Technology, Nihon University, Narashino, Chiba 275-8575 Japan; 2 National Institute for Fusion Science, National Institutes of Natural Sciences, Toki, Gifu 509-5292 Japan; 3 Center for Low-temperature Plasma Sciences, Nagoya University, Nagoya, 464-8601 Japan; 4 Interdisciplinary Graduate School of Engineering Sciences, Kyushu University, Kasuga, Fukuoka 816-8580 Japan

**Keywords:** Atomic and molecular interactions with photons, Plasma physics, Optical spectroscopy

## Abstract

Tunable diode laser absorption spectroscopy (TDLAS) is a valuable method for measuring particle flow velocities in plasma. However, conventional TDLAS using a plane-wave beam is sensitive only to the laser propagation direction. This limitation is particularly unfavorable for the observation of the particle transportation perpendicularly incident on the material in the plasma–material interaction. In this paper, we show for the first time that flow measurements perpendicular to the beam direction are possible by replacing the probe beam with an optical vortex beam. Because an optical vortex has a helical wavefront, particles moving in its field experience an azimuthal Doppler shift in addition to the translational Doppler shift. Assuming a uniform gas flow across the optical vortex, the azimuthal Doppler shift of the absorption spectrum observed in the beam cross-section varies sinusoidally in the azimuthal direction. The transverse flow velocity is derived from the amplitude of this sinusoidal variation. At transverse velocities above 70 m/s, the measurement errors are found to be less than 15%, with a mean absolute percentage error of less than 8%.

## Introduction

Laser spectroscopy, such as tunable diode laser absorption spectroscopy (TDLAS) and laser-induced fluorescence (LIF), is a non-intrusive plasma diagnostic method that can measure the velocity component of particles in a plasma^1–4^. In studies of plasma-surface interactions, such as sheath dynamics, heat flux to fusion reactor walls, and surface reactions in plasma processing, information on perpendicular particle flow in the boundary region is crucial; however, it is not accessible because the probe laser must be injected perpendicular to the surface to measure it^5,6^. This limitation in measurement direction is because Doppler spectroscopy using a plane wave is only sensitive to the velocity component along the wavevector.

On the other hand, a Laguerre-Gaussian (LG) beam has a helical wavefront, and Doppler spectroscopy using it is sensitive to translational, radial, and azimuthal velocity components^7–10^. In principle, the azimuthal Doppler shift can be used to measure rotational motion; however, this shift is much smaller than the translational Doppler shift and is therefore difficult to detect. For this reason, the azimuthal Doppler shift has been experimentally observed in systems without translational motion, such as rotating prisms or rotating plates^11–14^. It has also been detected for the first time in atomic systems as line broadening in the Hanle electromagnetically induced transparency (EIT) spectrum^15^. In this experiment, two superimposed LG beams with the same beam parameters other than the sign of the topological charge excite a three-level system under resonance conditions, and the azimuthal Doppler shift is detected as a broadening of the Hanle EIT spectrum. This method can detect the azimuthal Doppler shift with very high sensitivity; however, it is limited by the requirement that the two beams must be very precisely aligned, and that the EIT conditions must be fulfilled. In this paper, we report a spectroscopic method for detecting azimuthal Doppler shifts using TDLAS with an LG beam, and its application to gas-flow velocity measurements across the beam.

In the proposed method, we substitute the TDLAS probe beam with an optical vortex beam, and we refer to the method as optical vortex laser absorption spectroscopy (OVLAS)^16^. A collimated LG beam is used to measure the flow velocity ${U}_{x}$ in the direction transverse to the beam. The Doppler shift of the resonance condition for an atom in the collimated LG beam is given by^7,16^:

1 $$\delta _{{LG}} \approx - kv_{z} - \left( {\frac{\ell }{r}} \right)U_{x} \sin \phi$$

where ${\delta }_{LG}$ is the Doppler shift in the angular frequency, ${v}_{z}$ is the velocity component of the atom in the translational direction, $\ell$ is the topological charge, $r$ is the distance from the beam center, and ${U}_{x}$ is the uniform gas flow velocity in the $x$ direction. The first term is the translational Doppler shift, and the second is the azimuthal Doppler shift that is used to determine the transverse flow velocity. The radial Doppler shift is neglected because it is small under our experimental conditions. The azimuthal Doppler shift depends on $\ell$, $r$, and $\phi$. Therefore, the transverse velocity is obtained from the two-dimensional azimuthal Doppler shift distribution, as described in the next section.

## Results and discussion

**Figure 1:**
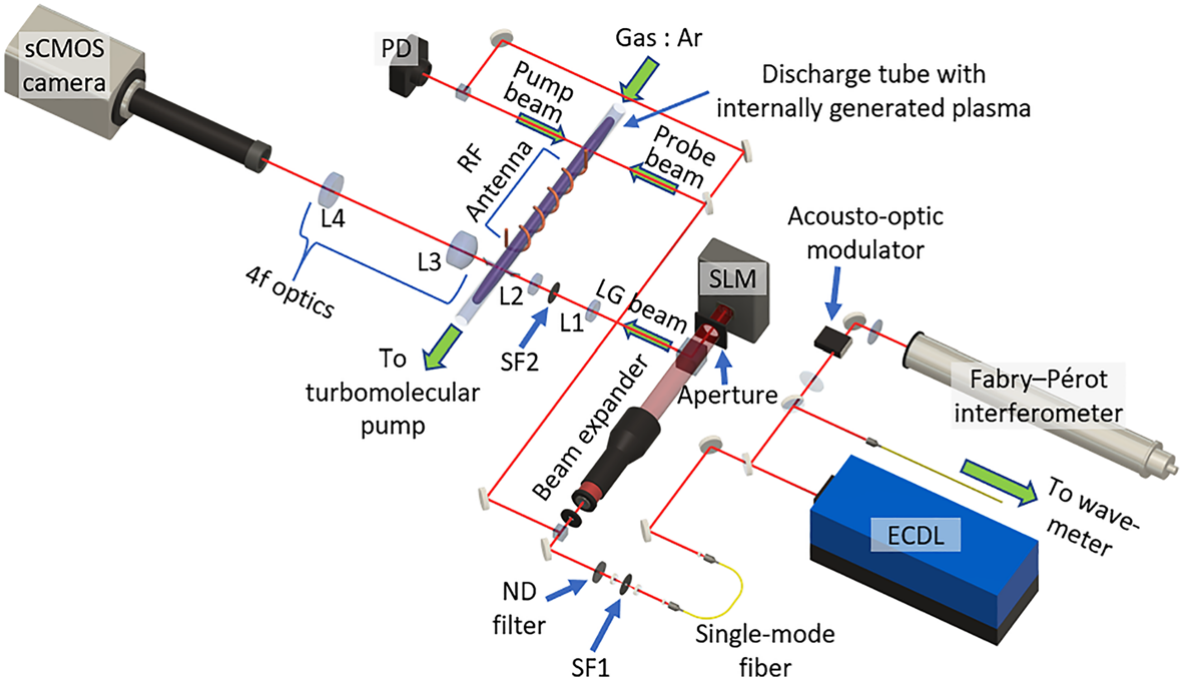
Transverse flow measurement system using optical vortex. The light source is an ECDL, and an optical vortex beam is generated by diffraction from the SLM. The diameter of the optical vortex beam is reduced to 1/6 by lenses L1 and L2 and is transversely injected into a discharge tube through a Brewster window. The transmitted light from the plasma is imaged by a camera with a 4f optical system composed of lenses L3 and L4. The discharge tube is evacuated by a turbo molecular pump and a scroll pump to generate flowing ICP plasma. The gas flow velocity is controlled by adjusting the gas flow rate using a mass flow controller.

The experimental setup used to validate the principle of transverse flow velocity measurement using OVLAS is shown in Fig. 1. A 697 nm external cavity diode laser (ECDL) is used as the light source, and the output light from the ECDL is mode-cleaned using a single-mode fiber and a spatial filter. The mode-cleaned Gaussian beam is incident on a spatial light modulator (SLM) that displays a computer-generated hologram (CGH), producing an optical vortex beam as diffracted light. The CGH is used to modulate the intensity and phase of the incident light in order to generate a high-quality optical vortex. In addition, a spatial filter is inserted to avoid contamination by neighboring orders of diffraction light^17–21^. The optical vortex beam is incident perpendicular to a discharge tube. Argon gas is introduced into the discharge tube, and an inductively coupled plasma (ICP) is generated by applying RF power to a loop antenna wound around the discharge tube. We observe the metastable state of argon, which is generated in the ICP and flows along the discharge tube, by absorption spectroscopy that corresponds to excitation from ${{}^{2}\mathrm{P}}_{3/2}^{0}4\mathrm{s}$ to ${{}^{2}\mathrm{P}}_{1/2}^{0}4\mathrm{p}$. A 4f optical system is focused on the plasma edge at the exit side. The intensity distribution of the optical vortex propagated through the plasma is transferred to an sCMOS camera. By synchronizing the camera shutter with the wavelength sweep of the ECDL, images are captured at each wavelength. The Doppler shift distribution in the beam cross-section is then obtained from the absorption spectra constructed from the intensity variation at each pixel in the wavelength sweep. The laser wavelength is calibrated by saturated absorption spectroscopy^22^. The gas flow velocity is also calibrated by performing TDLAS with a wavelength-calibrated Gaussian-mode laser with an optical path that is oblique to the gas flow (see Supplementary Information).

**Figure 2:**
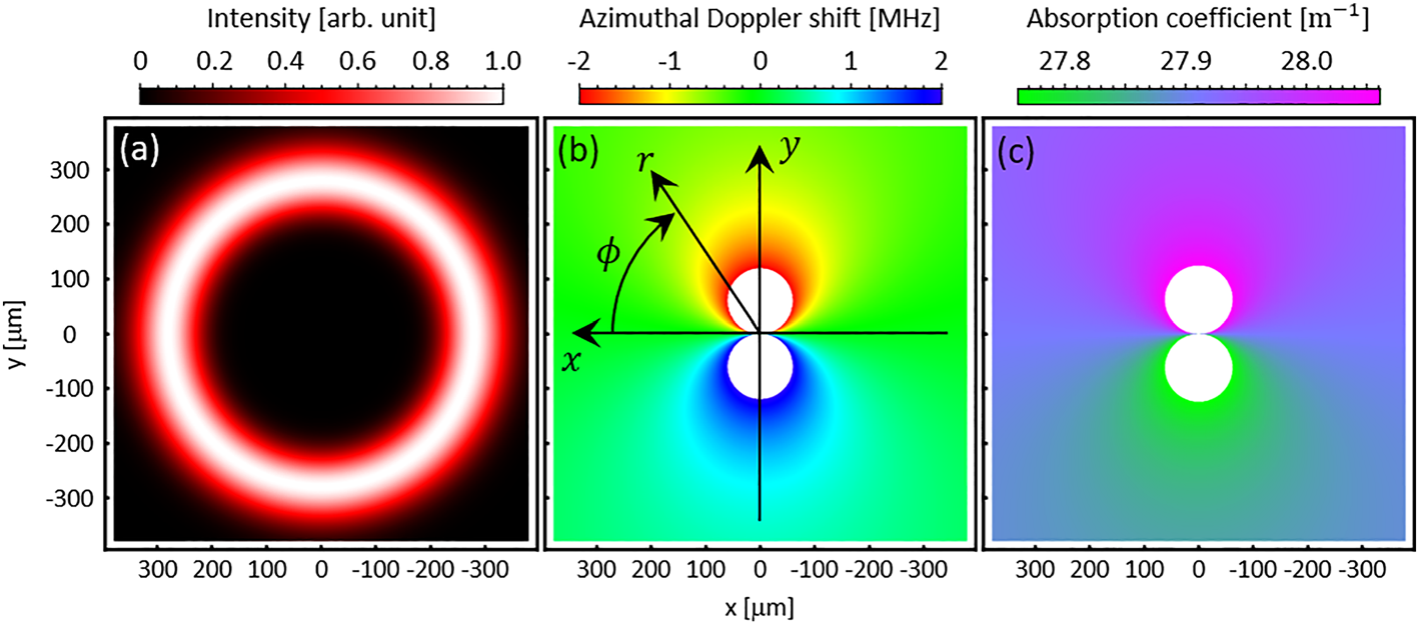
Numerical calculation results for (**a**) normalized intensity distribution for optical vortex beam, (**b**) azimuthal Doppler shift distribution, and (**c**) absorption coefficient distribution. The calculations are performed with parameters $\ell =+10$, ${U}_{x}=150$ m/s, and detuning = 360 MHz.

From Eq. (1), the Doppler shift consists of both translational and azimuthal components. Figure 2a and b show the theoretically expected intensity distribution and azimuthal Doppler shift distribution for the experimental conditions $\ell =+10$, ${U}_{x}=150$ m/s, respectively. The sign of the azimuthal Doppler shift is reversed in the upper and lower halves of the beam. As shown in Fig. 2b, the area near the center of the beam is theoretically advantageous for gas flow detection because the azimuthal Doppler shift is large. However, the phase distribution in the vicinity of the phase singularity is easily disturbed, making this an unsuitable position for measurement. Therefore, the high-intensity doughnut area is used to measure the flow velocity in the experiment. The absorption coefficient distribution calculated with the probe beam detuned to a higher-frequency shows that the optical vortex is always asymmetrically absorbed in a uniform flow (Fig. 2c). This inevitable generation of an anisotropic intensity distribution should be taken into account in the OVLAS analysis.

**Figure 3:**
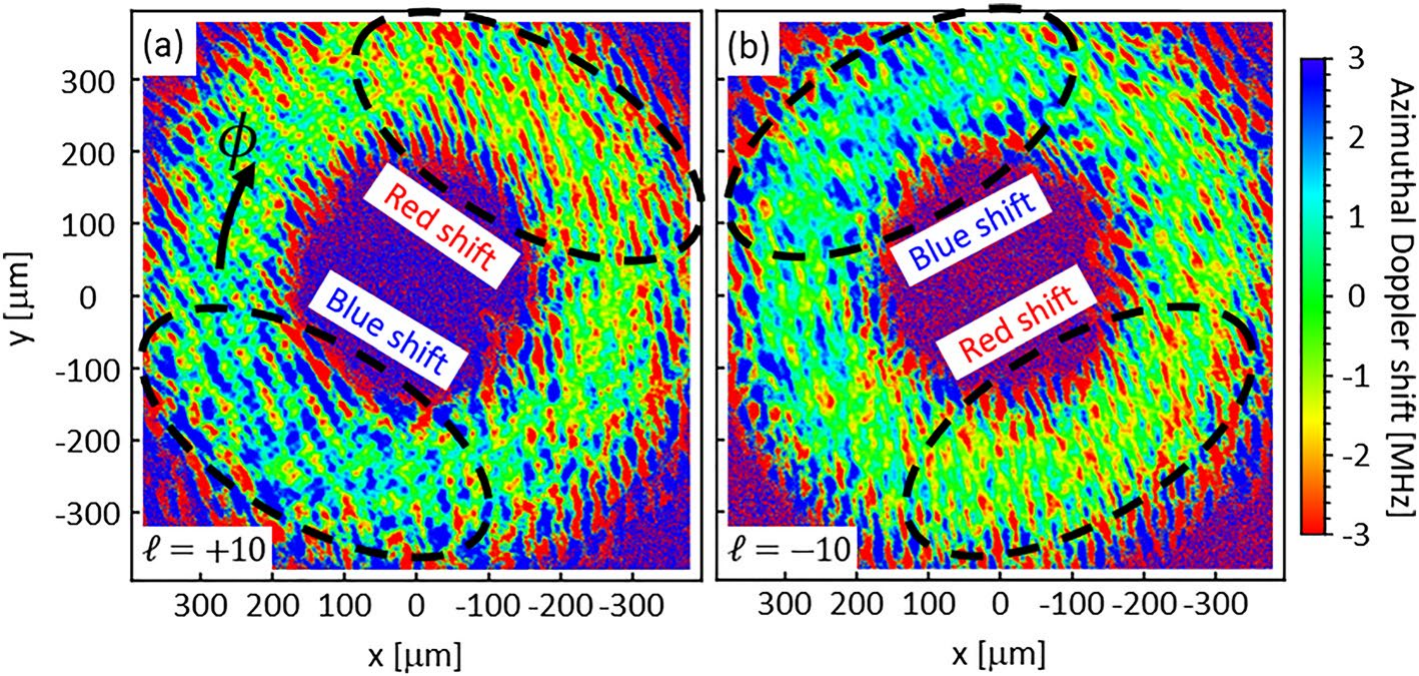
The azimuthal Doppler shift distribution observed by OVLAS. The distribution is rotated (**a**) clockwise for positive topological charge, (**b**) counterclockwise for negative topological charge.

Figure 3 shows the azimuthal Doppler shift distribution experimentally observed using an optical vortex with $\ell =\pm 10$ at ${U}_{x}=146$ m/s. The radius of the donut-shaped intensity peak is ${r}_{\mathrm{p}}$ = 262 µm. The observed Doppler shift distribution is rotated relative to the structure in Fig. 2b, and the direction of rotation depends on the sign of the topological charge. It should be noted that what is observed in the experiment is not the absorption coefficient distribution in the plasma, but it is the propagated intensity distribution for the optical vortex after absorption. As the optical vortex beam propagates in the transversely flowing plasma, the beam is absorbed anisotropically due to the azimuthal Doppler shift, as shown in Fig. 2c. Hamazaki et al. showed that the partially masked LG beam is rotated due to the Gouy phase shift while propagating in free space^23^. Similarly, the anisotropically absorbed structure is rotated by the Gouy phase shift; therefore, the azimuthal Doppler shift distribution derived from the absorption spectra also rotated. The experimental results are qualitatively consistent with our numerical results^16^.

**Figure 4:**
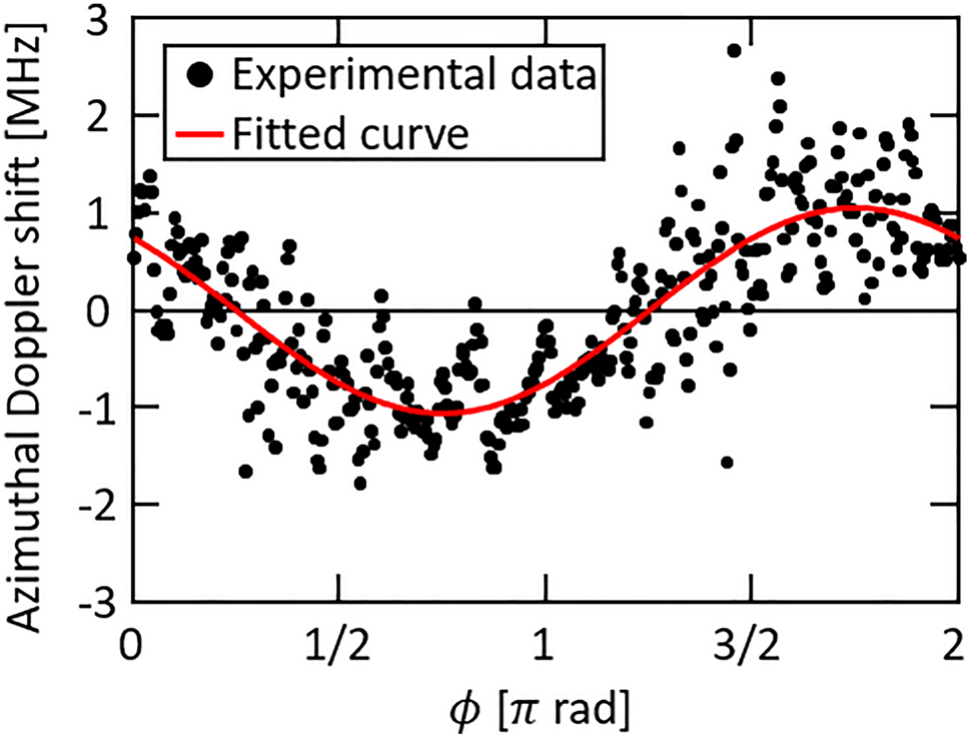
The azimuthal Doppler shift extracted in $\phi$ direction from Fig. 3a. The black symbols and the red line show the experimentally measured shift and the fitted sinusoidal curve, respectively. The transverse flow velocity is obtained from the amplitude of the sinusoidal variation of the azimuthal Doppler shift.

**Figure 5:**
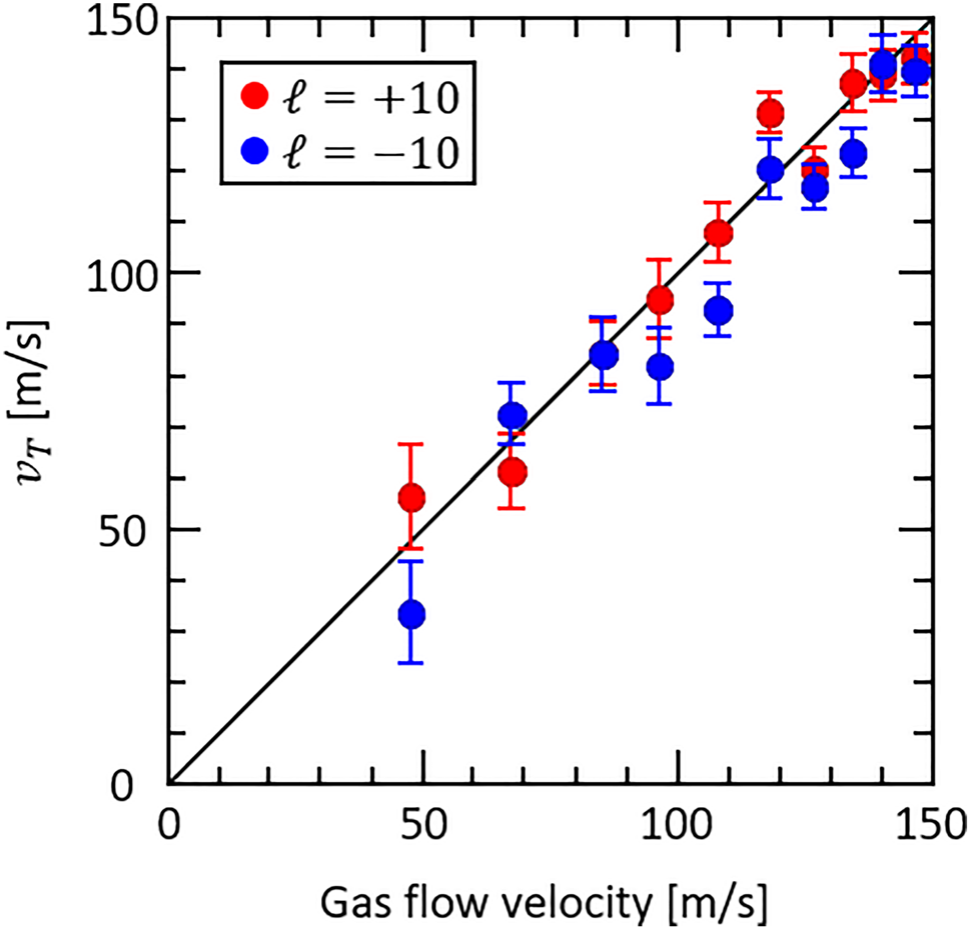
Measured transverse flow velocity for different gas flow velocities. The red and blue solid symbols show measurement results with $\ell =+10$ and $\ell =-10$, respectively.

Figure 4 shows the $\phi$ dependence of the azimuthal Doppler shift in Fig. 3a at the radial positions where the donut-shaped intensity is maximum, following subtraction of the translational Doppler shift. It should be emphasized that the transverse flow velocity is determined by the relative change in the azimuthal Doppler shift rather than the absolute laser wavelength. Since the azimuthal Doppler shift is inversely proportional to $r$ as expressed in Eq. (1), the azimuthal Doppler shift for each angle is converted to the value at ${r}_{\mathrm{p}}$ by multiplying by $r/{r}_{\mathrm{p}}$ and averaged. The resulting transverse flow velocity obtained from the amplitude of the sinusoidal variation was 145 ± 6 m/s, which is identical to the gas flow velocity within the fitting error. This result is the first demonstration of transverse flow measurement using an optical vortex. To evaluate the applicability of this method, OVLAS was performed with a gas velocity of 48 to 147 m/s, and the results are shown in Fig. 5. The horizontal axis represents the gas flow velocity calibrated by the saturated absorption measurement, and the vertical axis shows the transverse flow velocity ${v}_{T}$ measured by OVLAS. The errors at each measurement point were within 15% of the expected value, and the mean absolute percentage error (MAPE) was less than 8%, with the exception of the case with the lowest gas velocity. These results show that OVLAS can measure the transverse velocity with high accuracy (see Supplementary Information).

## Conclusion

We have developed the OVLAS method that substitutes the traditional TDLAS probe beam with an optical vortex. An optical vortex beam was injected perpendicular to the gas flow, and the profiles of the absorbed beam were recorded with a sCMOS camera synchronized with the laser wavelength sweep. The Doppler spectra were constructed at each pixel to obtain the azimuthal Doppler shift distribution. As predicted by the theory, the azimuthal Doppler shift varies sinusoidally in the $\phi$ direction, and the transverse flow velocity was derived from the amplitude of the sinusoidal variation. This result is the first demonstration of spectroscopic transverse flow measurements of an atomic system using the azimuthal Doppler shift. The MAPE for the measurements was less than 8% for flow velocities above 70 m/s. In future work, we plan to apply OVLAS to observe particle transport in the boundary region between a plasma and a material.

## Methods

### Calibration of sweep frequency of tunable laser

We used a Fabry–Perot interferometer (FPI) calibrated by an acousto-optic modulator (AOM) to accurately measure the Doppler shift. In Fig. 1, the output light from the ECDL is split and injected into the FPI via the AOM. The frequency of the first-order diffracted light in the AOM is shifted by 80 MHz relative to that of the zeroth-order diffracted light. Therefore, when both zero-order and first-order diffracted light are introduced into the FPI, spectra with a frequency separation of 80 MHz are obtained. This known spectral shift is used to calibrate the free spectral range (FSR) of the FPI. While sweeping the wavelength of the ECDL, the spectrum is detected by the FPI with the FSR interval, allowing the sweep frequency to be calibrated.

### Supplementary Information

Supplementary Information.

## Data Availability

The datasets used in the current study are available from the corresponding author upon reasonable request.
