# Supplementary material for: Enhancement of Doppler spectroscopy to transverse direction by using optical vortex
Source: Sci Rep. 2023 Sep 16;13:15400. doi: 10.1038/s41598-023-42517-z (PMC10505230; doi:10.1038/s41598-023-42517-z)
Supplement: Supplementary file 1 — Supplementary Information. [file 41598_2023_42517_MOESM1_ESM.pdf]

Supplementary Information:

Enhancement of Doppler spectroscopy to transverse direction by using optical vortex

Hiroki Minagawa[1], Shinji Yoshimura[2,3], Kenichiro Terasaka[4],and Mitsutoshi Aramaki[1,*]

[1] *College of Industrial Technology, Nihon University, Narashino, Chiba, 275-8575, Japan*

[2] *National Institute for Fusion Science, National Institutes of Natural Sciences, Toki, Gifu 509-5292, Japan*

[3] *Center for Low-temperature Plasma Sciences, Nagoya University, Nagoya 464-8601, Japan*

[4] *Interdisciplinary Graduate School of Engineering Sciences, Kyushu University, Kasuga, Fukuoka 816-8580, Japan*

[*]aramaki.mitsutoshi@nihon-u.ac.jp

Calibration of gas flow velocity

The gas flow velocity in the discharge tube was calibrated by simultaneously performing conventional TDLAS and saturated absorption spectroscopy (SAS), as shown in Fig. S1. Since these measurements were performed with a plane-wave-like Gaussian beam, the TDLAS probe beam was injected obliquely into the gas flow to obtain the translational Doppler shift due to the gas flow as a projection onto the wavenumber vector. SAS was conducted along the same optical path as the OVLAS probe beam, and the resonance absorption frequency was obtained from the Lamb dip in the saturated absorption spectrum [S1]. Figures S2(a) and 2(b) show the saturated absorption spectrum and the absorption spectrum observed with a 300-ccm gas flow, respectively. The absorption spectrum is shifted by 155 MHz from the resonance frequency, and the gas flow velocity is determined to be 117 m/s considering that the incidence angle of the probe beam to the gas flow was 0.39 rad. The detuning frequency for the absorption spectrum from the resonance frequency was calibrated by FPI as described in Methods. This experiment was conducted under a constant pumping velocity, which means that in addition to changes in the flow velocity, the pressure varies with gas flow rate. Furthermore, the product of pressure and flow velocity is proportional to the gas flow rate. The results of gas velocity measurements at various gas flow rates, ranging from 50 to 500 ccm, are presented in Fig. S3. The relationship between the gas flow rate and the flow velocity is nonlinear because the gas pressure changes simultaneously.

Translational Doppler shift component in OVLAS

In our experimental system, the OVLAS probe beam is injected almost perpendicular to the gas flow; however, this alignment is not perfect. Therefore, the observed Doppler shift also includes a small translational Doppler shift component, which is excluded in Fig.3 to show the azimuthal Doppler shift distribution. The translational Doppler shift is obtained from the offset of the ϕ-directional variation of the Doppler shift distribution. Figure S4 shows the dependence of the translational Doppler shift on the gas flow velocity. The translational Doppler shift varied linearly with gas flow velocity, as expected, and from this relation, the deviation of the probe beam from the vertical direction was estimated to be 0.027 radians (1.55°). While evaluation of the translational Doppler shift depends on the calibrated laser wavelength, measurement of the transverse Doppler shift does not require such a calibration. Thus, in the OVLAS experiment, the laser wavelength was calibrated by saturation absorption spectroscopy only as a supplementary measure, resulting in a relatively large MAPE of approximately 20% for the translational Doppler shift.

OVLAS measurement errors

In this study, we evaluated the OVLAS measurement errors in relation to the calibrated gas flow velocity using the measurement system shown in Fig. S1; the results are given in Table 1. The transverse flow velocities, denoted by v_+ and v_- for optical vortex beams with $\ell = +10$ and $\ell = -10$, respectively, were measured for gas velocities (v_g) ranging from 48 to 147 m/s, respectively. Here, Δ represents the difference between v_+ and v_- from v_g. Excluding the case for v_g = 48 m/s, the value of Δ was distributed below 15%. The MAPE for v_+ and v_- was 5% and 8%, respectively. While the evaluation of the translational Doppler shift in Fig. S4 depends on the calibrated laser wavelength, the transverse flow velocity is determined only by the relative change in the Doppler shift in the azimuthal direction, leading to reduced errors.

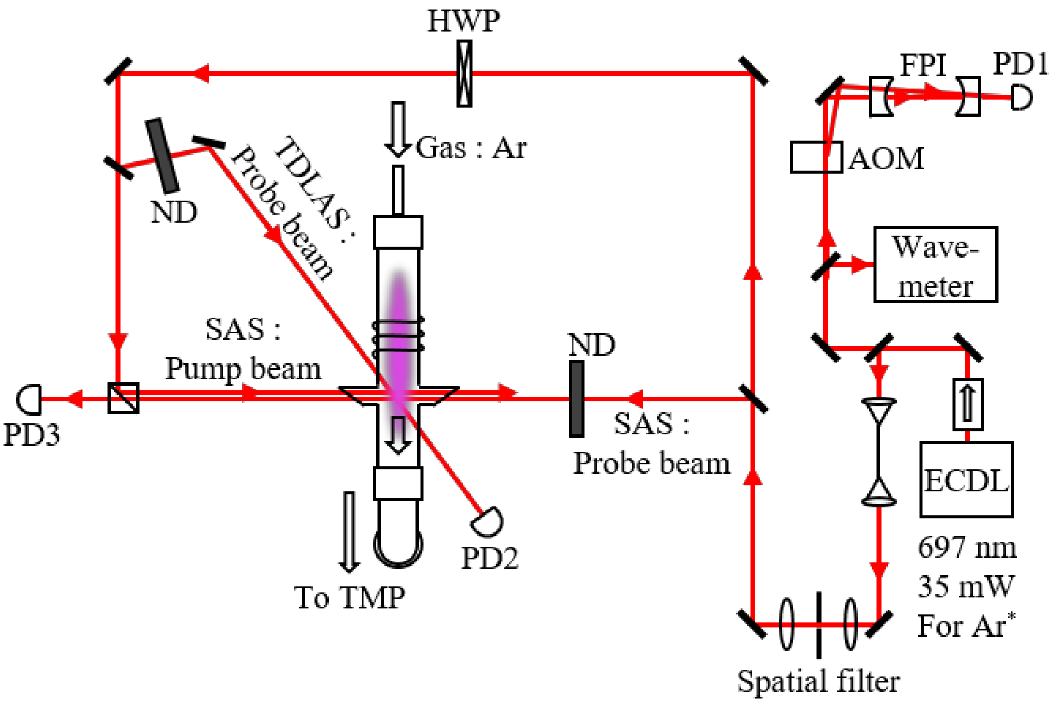

Figure S1. Gas flow velocity measurement using TDLAS and SAS.

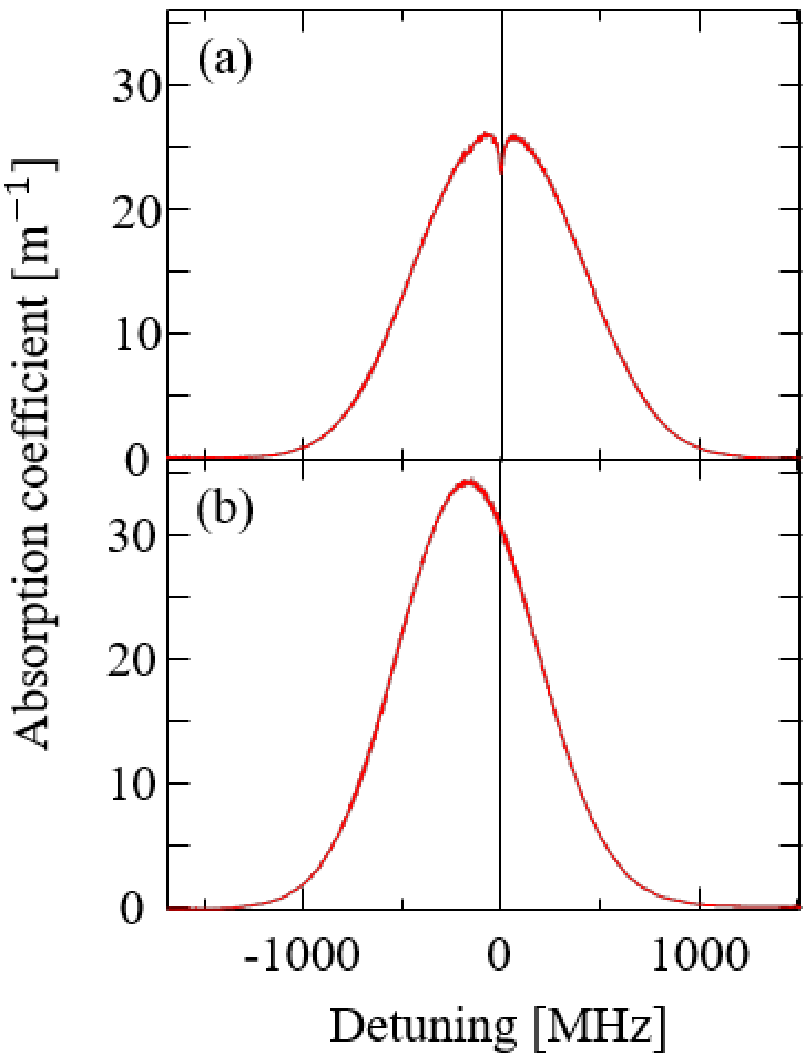

Figure S2. (a) Saturated absorption spectrum. (b) Absorption spectrum.

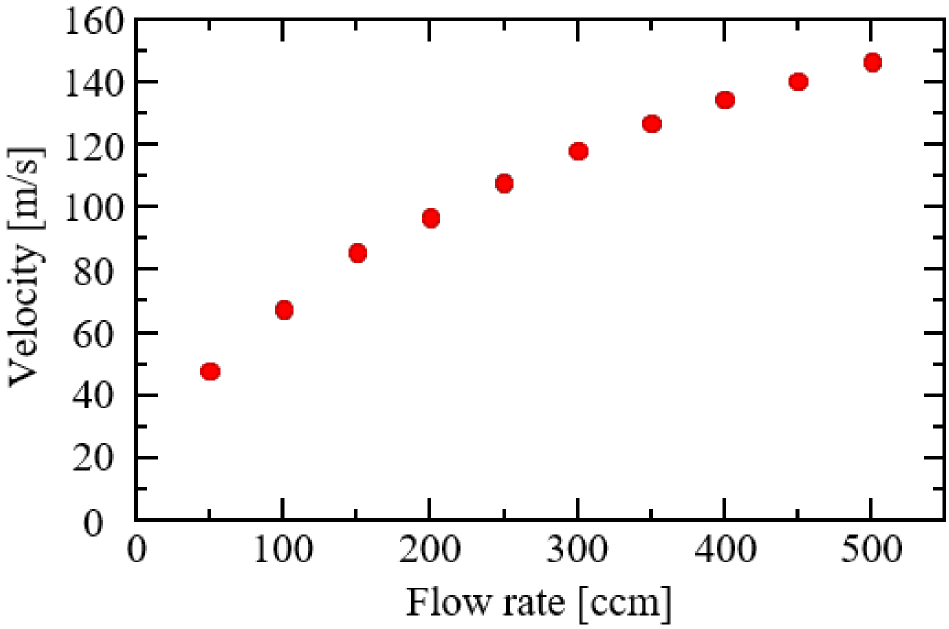

Figure S3. Dependence of gas flow velocity on gas flow rate.

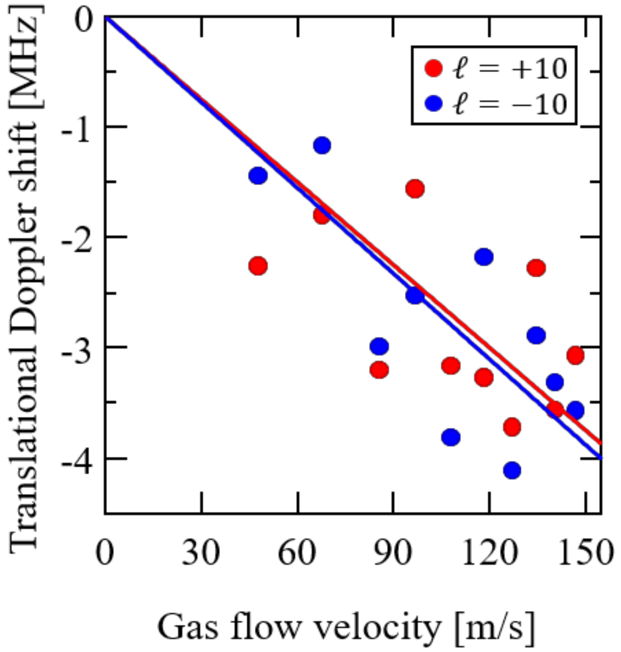

Figure S4. Dependence of translational Doppler shift on gas flow velocity.

Table S1. OVLAS measurement errors.

v_g [m/s]	v_+ [m/s]	Δ [%]	v_- [m/s]	Δ [%]
48	56±11	17	33±11	32
67	61±8	9	72±7	8
85	84±7	2	84±8	2
96	95±8	2	82±8	15
108	108±6	0	93±6	14
118	132±4	12	121±6	3
127	120±5	6	117±5	8
134	138±6	3	124±5	8
140	139±5	1	141±6	1
147	144±6	3	140±6	5

Reference

[S1] Wolfgang, D. Laser spectroscopy vol. 2 experimental techniques 5th Edition 96-99 (Springer-Verlag, Berlin, Heidelberg, 2015).
